# X-linked severe combined immunodeficiency due to *IL2RG* p.V223F variant: clinical evidence that support its pathogenicity- a case report

**DOI:** 10.3389/fimmu.2026.1776191

**Published:** 2026-04-14

**Authors:** Bricia Melissa Gutiérrez-Zepeda, Juan Carlos Lona-Reyes, Mario Ernesto Cruz-Muñoz, Antonio Quintero-Ramos, Carlos Torres-Lozano, Beatriz Bayardo-Gutierrez, Alejandro Barrón-Balderas, Sergio D. Rosenzweig, Jennifer Stoddard, Julie Niemela, María Enriqueta Núñez-Núñez

**Affiliations:** 1 Doctorado en Genética Humana, Departamento de Biología Molecular y Genómica, Centro Universitario de Ciencias de la Salud, Universidad de Guadalajara, Guadalajara, Mexico; 2 Instituto de Investigación en Inmunología, Departamento de Fisiología, Centro Universitario de Ciencias de la Salud, Universidad de Guadalajara, Guadalajara, Mexico; 3 Departamento de Infectología, Nuevo Hospital Civil de Guadalajara “Dr. Juan I. Menchaca”, Guadalajara, Mexico; 4 Facultad de Medicina, Universidad Autónoma del Estado de Morelos, Cuernavaca, Mexico; 5 Unidad de Investigación Biomédica 02, Unidad Médica de Alta Especialidad (UMAE), Hospital de Especialidades, Centro Médico Nacional de Occidente, Instituto Mexicano de Seguro Social (IMSS), Guadalajara, Mexico; 6 Ex Jefe del departamento de Inmunología Clínica y Alergia, Hospital de Especialidades, Centro Médico Nacional de Occidente, Instituto Mexicano de Seguro Social (IMSS), Guadalajara, Mexico; 7 Departamento de Alergia e Inmunología Clínica Pediátrica, Nuevo Hospital Civil de Guadalajara “Dr. Juan I. Menchaca”, Guadalajara, Mexico; 8 Departamento de Clínicas de la Reproducción Humana, Crecimiento y Desarrollo Infantil, Centro Universitario de Ciencias de la Salud, Universidad de Guadalajara, Guadalajara, Mexico; 9 Immunology Service, Department of Laboratory Medicine, National Institutes of Health (NIH) Clinical Center, NIH, Bethesda, MD, United States

**Keywords:** c.667 G>T, case report, *IL2RG* gene, p.V223F, rs2092258151, severe combined immunodeficiency, X-linked severe combined immunodeficiency

## Abstract

**Introduction:**

Severe combined immunodeficiency (SCID) comprises a group of life-threatening inborn errors of immunity (IEI) characterized by profound T-cell deficiency, frequently accompanied by impaired B cell and natural killer (NK) cell function. X-linked SCID (X-SCID), caused by pathogenic variants in *IL2RG*, accounts for approximately 30% of all SCID cases.

**Case presentation:**

We describe two male siblings born to consanguineous parents with a family history of early sibling deaths due to severe infections. Patient 1 was a 9-month-old boy who developed persistent cough, chronic diarrhea, and subcutaneous nodules following Bacillus *Calmette–Guérin* (BCG) vaccination. He was subsequently diagnosed with disseminated BCG infection with concomitant *Salmonella* co-infection. Immunological evaluation revealed a T−B+NK− phenotype. Despite intensive antimicrobial treatment, he died of septic shock at 12 months of age. Patient 2, a one-month-old boy, was evaluated early in life because of family history. Immunophenotyping demonstrated absent T cells, normal B cells, and reduced NK cells, along with the absence of a thymic shadow on chest radiography. Next-generation sequencing identified a hemizygous *IL2RG* c.667G>T (p.V223F). He received antimicrobial prophylaxis and immunoglobulin replacement therapy; however, he developed disseminated adenovirus infection and died at 8 months of age. Molecular Findings: *In silico* analyses (UniProt, HOPE, dbNSFP) consistently supported the pathogenic effect of the variant *IL2RG* p.V223F. Based on this evidence, we propose that its current classification as “likely pathogenic” should be updated to “pathogenic”.

**Discussion:**

These cases underscore the challenges faced by patients with SCID when timely access to curative therapy is not available. They also highlight the importance of readily accessible but highly informative diagnostic clues, such as the absence of a thymic shadow on chest radiography and the occurrence of severe complications following BCG vaccination. Conclusions: This report expands the known genotypic and phenotypic spectrum of SCID and reinforces the critical need for early diagnosis, appropriate genetic counseling in consanguineous families, and equitable access to newborn screening programs and curative treatments, including hematopoietic stem cell transplantation and gene therapy, in order to improve survival outcomes.

## 1 Introduction

Severe combined immunodeficiency (SCID) represents a group of inborn errors of immunity (IEI), characterized by impaired T and B and/or NK lymphocyte development and life-threatening susceptibility to infections (1). The most prevalent form is X-linked SCID (X-SCID), caused by mutations in the IL2RG gene (2), which encodes the common gamma chain (γc), shared by multiple interleukin receptors (IL-4, IL-7, IL-9, IL-15, and IL-21). Defective γc signaling typically results in a T^−^B^+^NK^−^ immunophenotype (3), leading to profound cellular and humoral immune dysfunction (4, 5).

While the genetic landscape of X-SCID is well-documented, phenotypic variability remains a challenge for clinical management and variant classification. Mutations in *IL2RG* can exhibit variable expressivity, where the same genotype results in different degrees of immune impairment (6). Specifically, the missense variant c.667G>T (p.V223F) has been previously reported; however, its clinical impact and classification require further clarification based on real-world outcomes (7).

This report addresses a critical knowledge gap regarding the severity and progression of the p.V223F variant. We describe the disease course of two siblings from a consanguineous family who exhibited an aggressive phenotype with fatal outcomes. By documenting severe complications following BCG vaccination and the rapid clinical deterioration linked to this variant, our findings provide additional evidence supporting the reclassification of p.V223 from “likely pathogenic” to “pathogenic” in accordance with the American College of Medical Genetics and Genomics (ACMG) criteria (8).

### 1.1 Case presentation

#### 1.1.1 Patient 1

**Figure 1:**
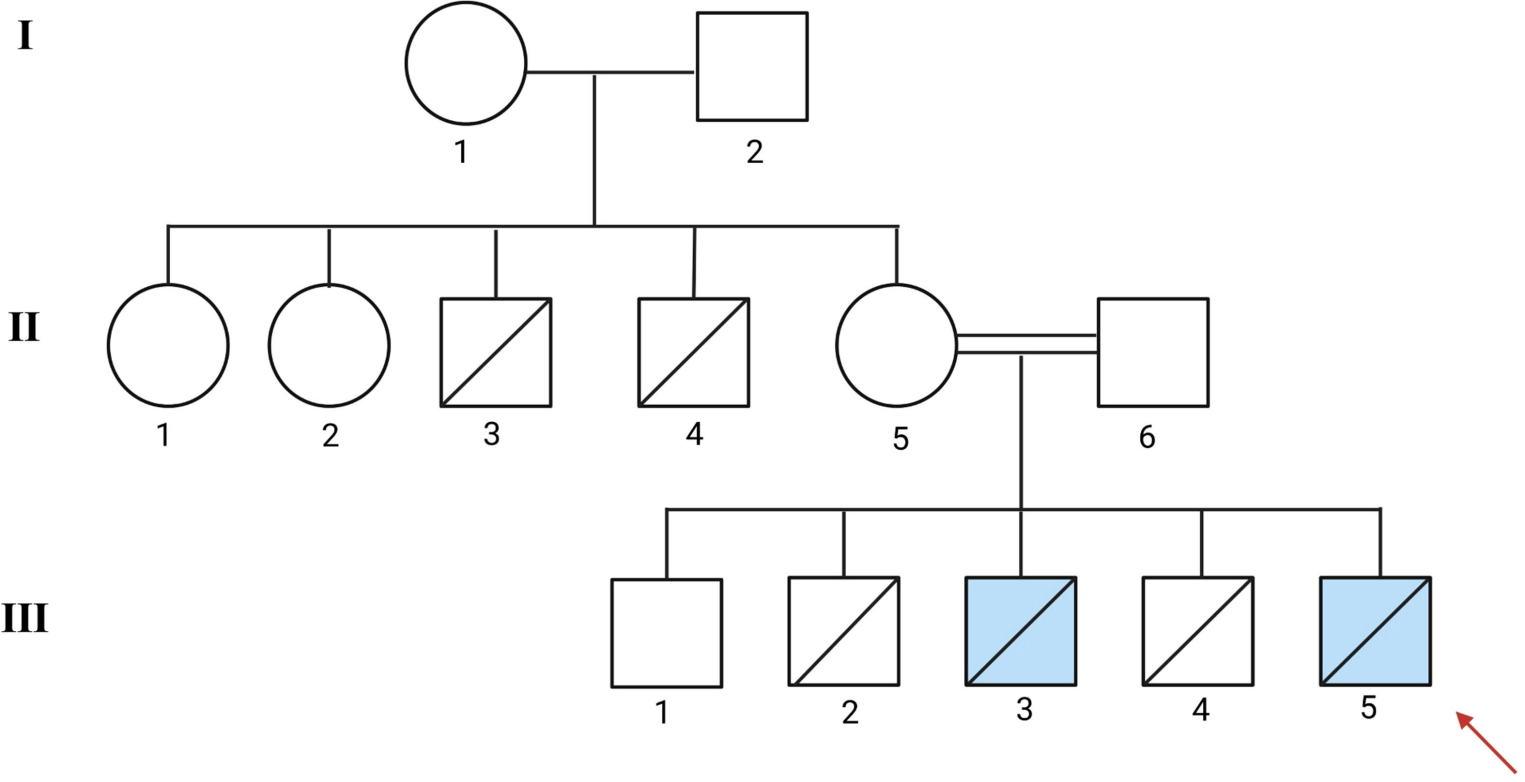
Genealogy of a consanguineous family with multiple cases of early-onset immunodeficiency. The figure illustrates the recurrent early mortality among male siblings. The affected individuals are represented by blue squares, and the individual analyzed by sequencing is indicated with a red arrow. The inheritance pattern suggests an X-linked disorder. Created by BioRender. Bravo, K (2025). https://BioRender.com/0xjwll7.

A 9-month-old boy was admitted to our hospital with a severe pulmonary infection accompanied by subcutaneous nodules He was the third child of consanguineous parents, (**Figure 1**:II-5-6), and was born at term after an uncomplicated vaginal delivery, his birth weight was 2800 g and his length of 50 cm. The family history was notable for early deaths due to infectious diseases on the maternal side. Two maternal uncles (**Figure 1**: II-3 and II-4) died at 6 and 8 months of age, respectively, from diarrheal illnesses, whereas two maternal aunts survived (**Figure 1**: II-1 and II-2). Among the patient’s siblings, the eldest brother, a healthy 15-year-old boy (**Figure 1**: III-1), is alive and well. A second brother (**Figure 1**: III-2) died at 12 months of age from septic shock.

**Figure 2:**
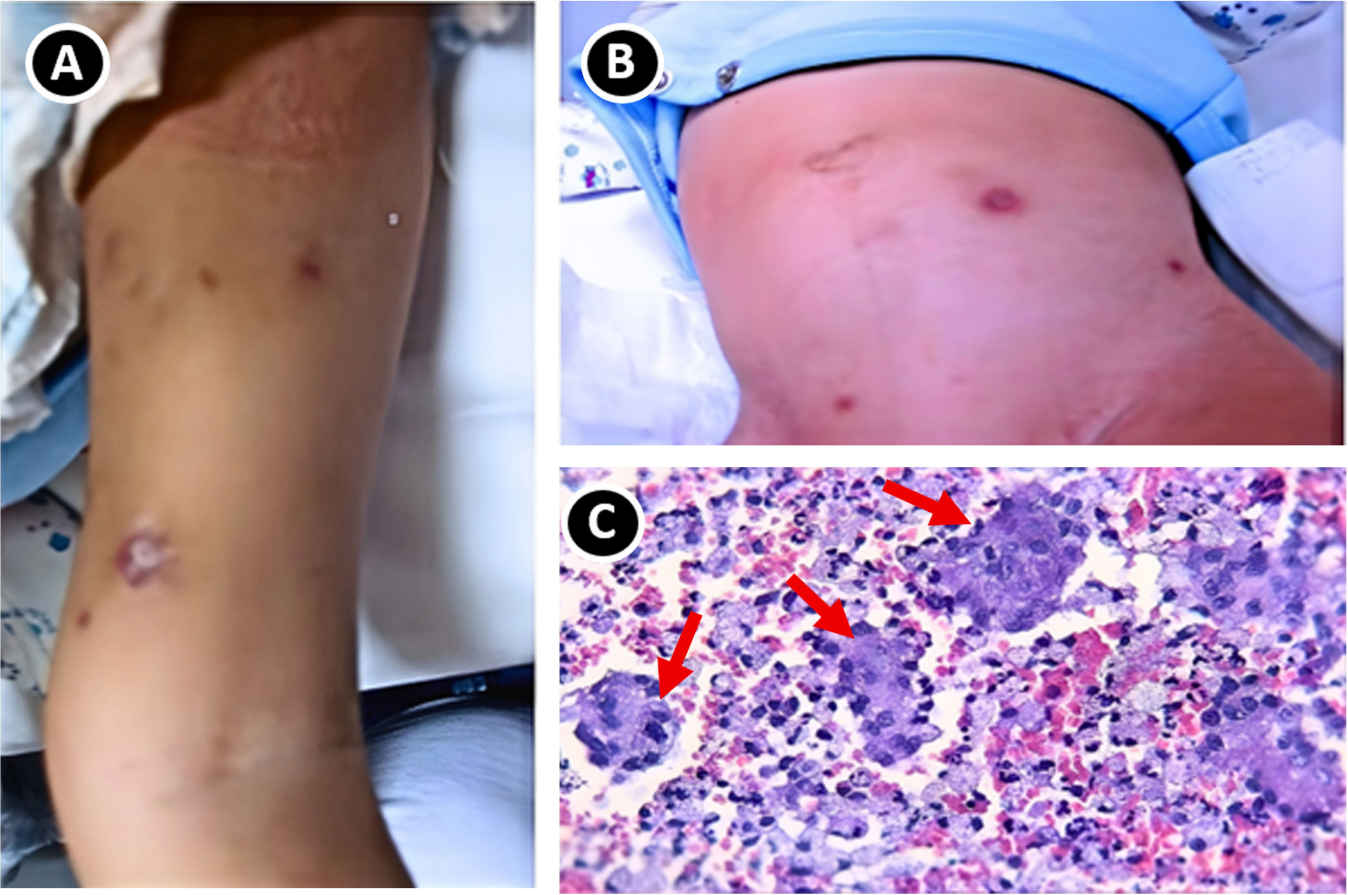
Clinical presentation and histopathology of skin lesions in Patient 1. **(A, B)**: **(A)** Arm and **(B)** abdomen showing several hyperpigmented subcutaneous nodules. **(C)** Histopathological image stained with 40X hematoxylin and eosin showing dermal areas with chronic granulomatous inflammation composed of multinucleated giant cells with abundant foamy cytoplasm and cellular debris within (red arrows), overlying a necrotic background.

The patient received the *Bacillus Calmette–Guérin* (BCG) vaccination in the left upper arm at birth (Danish strain 1331). At one month of age he developed a persistent cough. By three months, he exhibited chronic diarrhea, progressive cough, and failure to thrive with associated weight loss. At five months of age, the BCG inoculation site became swollen and began to drain purulent material. In addition, multiple hyperpigmented subcutaneous nodules appeared on the lower extremities, abdomen, and right arm. Multiple firm, non-tender subcutaneous nodules measuring 0.5–1.0 cm were observed on the arms and abdomen (**Figures 2A, B**). At 9 months of age, a biopsy of one of the subcutaneous lesions demonstrated chronic granulomatous inflammation suggestive of mycobacterial infection (**Figure 2C**). A complete blood count revealed cytopenias, prompting referral to our tertiary care center for further evaluation. Upon hospital admission, the patient weighed 6.5 kg (P<1), measured 65 cm (P<1), and exhibited hipoactivity with tachypnea and signs of increased work of breathing, including intercostal and xiphoid retractions. Lung auscultation demonstrated vesicular breath sounds with coarse characteristics, and cardiac examination revealed a grade II holosystolic murmur.

Initial laboratory evaluation showed a white blood cell count 5.64×109/L (reference range, 6×109/L-17×109/L); lymphocyte count 0.14×109/L (4×109/L-13.5×109/L); hemoglobin 13.9 g/dL (10.5 g/dL-14.1 g/dL), platelet count 100×109/L (140×109/L-450×109/L). Immunological assessment demonstrated reduced serum immunoglobulin (Ig) levels, with IgG 173 mg/dL (217–984 mg/dL); IgE: 0.7 UI/mL (0.76-7.31 UI/mL) with undetectable IgA and IgM. Lymphocyte subset analysis confirmed profound T-cell lymphopenia, with total T cells of 489 cells/µl (2500–6100); Further characterization showed CD3^+^ cells at 14 cells/µL (1500–2900 cells/µL); CD4^+^cells at 7 cells/µL (1000–2100 cells/µL); CD8^+^ cells at 6 cells/µL (700–1100 cells/µL); B cells CD19^+^ were relatively preserved at 440 cells/µL (500–1500 cells/µL), whereas NK cells CD 56^+^ were markedly reduced at 11 cells/µL (250–600 cells/µL) (**Table 1**).

**Table 1:** Immunological findings in Patient 1 (9 months of age) and Patient 2 (1 month of age).

Variable	Patient 1 (9 months of age)	Reference ranges	Patient 2 (one month of age)	Reference ranges
Serum immunoglobulins				
IgG (mg/dL)	173	217 - 984	632	310 - 852
IgM (mg/dL)	0	34 - 126	15.3	4.2 - 26
IgA (mg/dL)	0	11 - 90	< 5	3.5 - 67
IgE (UI/mL)	0.7	0.76 - 7.31	0.1	0.7 - 2.1
Lymphocyte subsets				
Lymphocytes cells/µL	489	2500 - 6100	1560	3080 - 6748
CD3+ (cells/µL)	14	1500 - 2900	0	2537 - 5785
CD4+ (cells/µL)	7	1000 - 2100	0	1971 - 4375
CD8+ (cells/µL)	6	700 - 1100	5	539 - 1752
CD19+ (cells/µL)	440	500 - 1500	1504	430 - 3300
CD16+/CD56+ (cells/µL)	11	250 - 600	24	289 - 690

Review of histological slides confirmed the presence of acid-fast bacilli (AFB),. Stool culture yielded *Salmonella* spp. Active cytomegalovirus (CMV) infection was documented by positive pp65 antigenemia. Culture of the biopsy specimen grew *Mycobacterium tuberculosis* with resistance to pyrazinamide. The patient was therefore started on antimicrobial therapy targeting tuberculosis, salmonellosis, and CMV infection.

**Figure 3:**
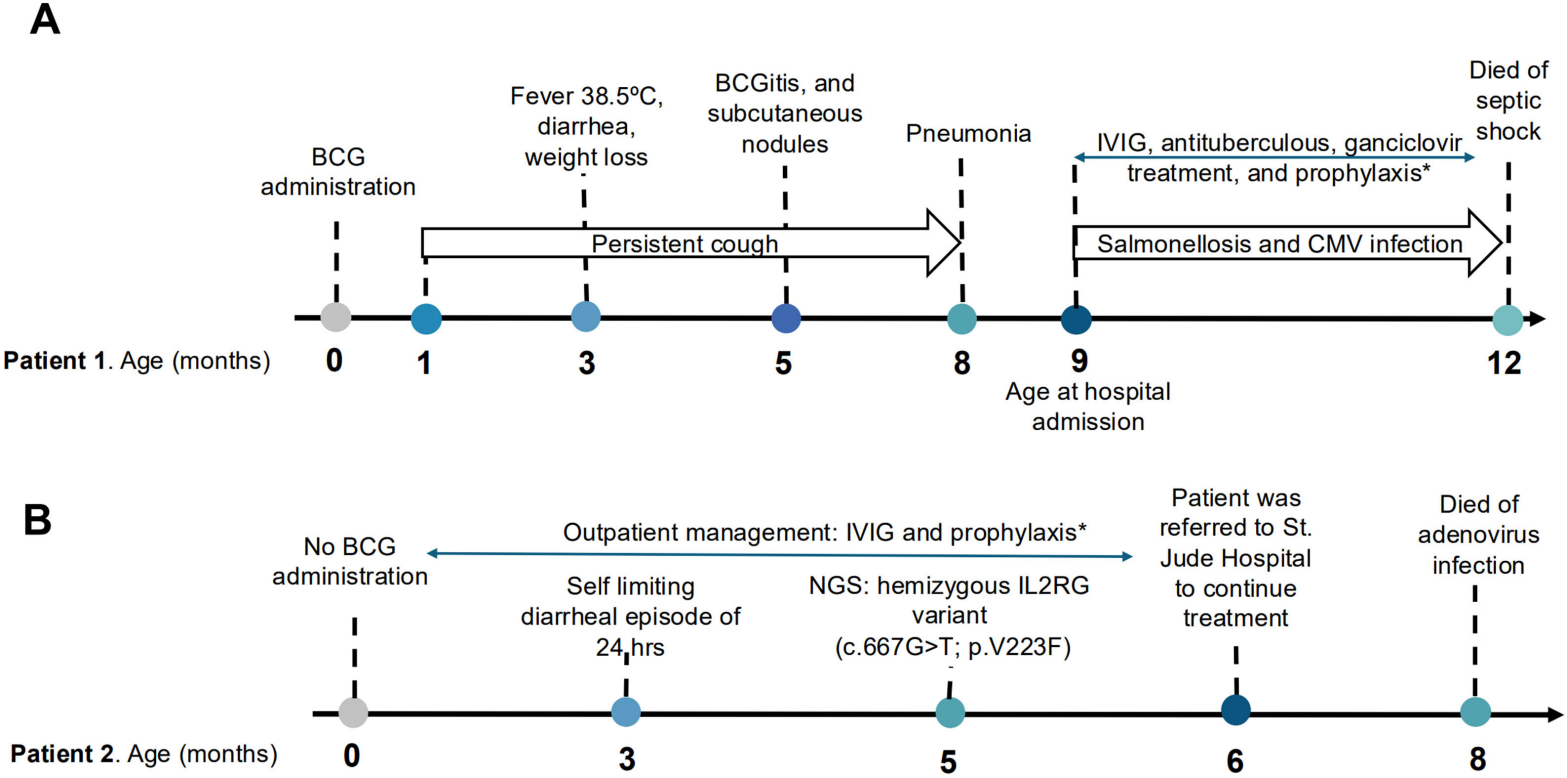
Chronological representation of clinical manifestations, infections, diagnostic findings, and treatments. In **(A)** Patient 1 and **(B)** Patient 2. Blue arrows indicate ongoing therapies. IVIG, intravenous immunoglobulin; NGS, Next Generation Sequencing; prophylaxis* included trimethoprim-sulfametoxazol, antiviral agents and fluconazole.

Based on the clinical presentation and immunological findings, the patient was diagnosed with SCID exhibiting a T^−^B^+^NK^−^ immunophenotype. He received antituberculous therapy consisting of rifampicin (10 mg/kg/day), isoniazid (10 mg/kg/day), and ethambutol (30 mg/kg/day). Additional treatment included antifungal therapy with fluconazole (6 mg/kg/day), antibacterial coverage with ciprofloxacin (30 mg/kg/day), and ticarcillin/clavulanic acid (200 mg/kg/day); antiviral therapy with ganciclovir (10 mg/kg/day); and prophylaxis with trimethoprim-sulfamethoxazole (TMP–SMX) (5 mg/kg every third day) Intravenous immunoglobulin (IVIG) replacement therapy was administered at (800 mg/kg/month). A hematopoietic stem cell transplantation (HSCT) protocol was initiated; however, the patient died at 12 months of age due to septic shock. The timeline of events is summarized in **Figure 3A**. Genetic diagnostic testing could not be performed.

#### 1.1.2 Patient 2

An 8-month-old boy, the younger brother of patient 1, was the product of the fifth pregnancy (**Figure 1**:III-5). He was born after an uncomplicated pregnancy and delivery, with a birth weight of 3,325 g and a length of 51 cm. Prior to his birth, a male fetus from a previous pregnancy had died *in utero* (**Figure 1**:III-4). Given the family history, no immunizations were administered. Breast feeding was discontinued because the mother tested positive for CMV, while the patient tested negative.

Growth and developmental milestones were appropriate for age, and the physical examination was unremarkable. At one month of age, he weighed 4,075 g (P34), height 53 cm (P24). Complete blood count demonstrated a white blood cell counts of 6×10^9^/L (reference values 4×109/L-19.5×109/L); and an absolute lymphocyte count of 1.56×109/L (2.5-16.5×109/L); hemoglobin was 11.7 g/dL (13–20 g/dL), and the platelet count was 535×109/L (150-400×109/L). Immunological evaluation showed serum immunoglobulin levels of IgG: 632 mg/dL (310–852 mg/dL), IgM: 15.3 mg/dL (4.2–26 mg/dL), IgA: <5 mg/dL (3.5–67 mg/dL); and IgE: 0.1 UI/mL (0.7-2.1 UI ml). Lymphocyte subset analysis revealed profound T-cell lymphopenia, with CD3^+^ and CD4^+^ cells undetectable (0 cells/µL, reference values 2537–5785 and 1971–4375 cells/µL, respectively), B cells CD19^+^ were within the normal range at 1504 cells/µL (430–3300 cells/µL), and reduced NK cells CD56^+^ 24 cells/µL (289–690 cells/µL) (**Table 1**).

**Figure 4:**
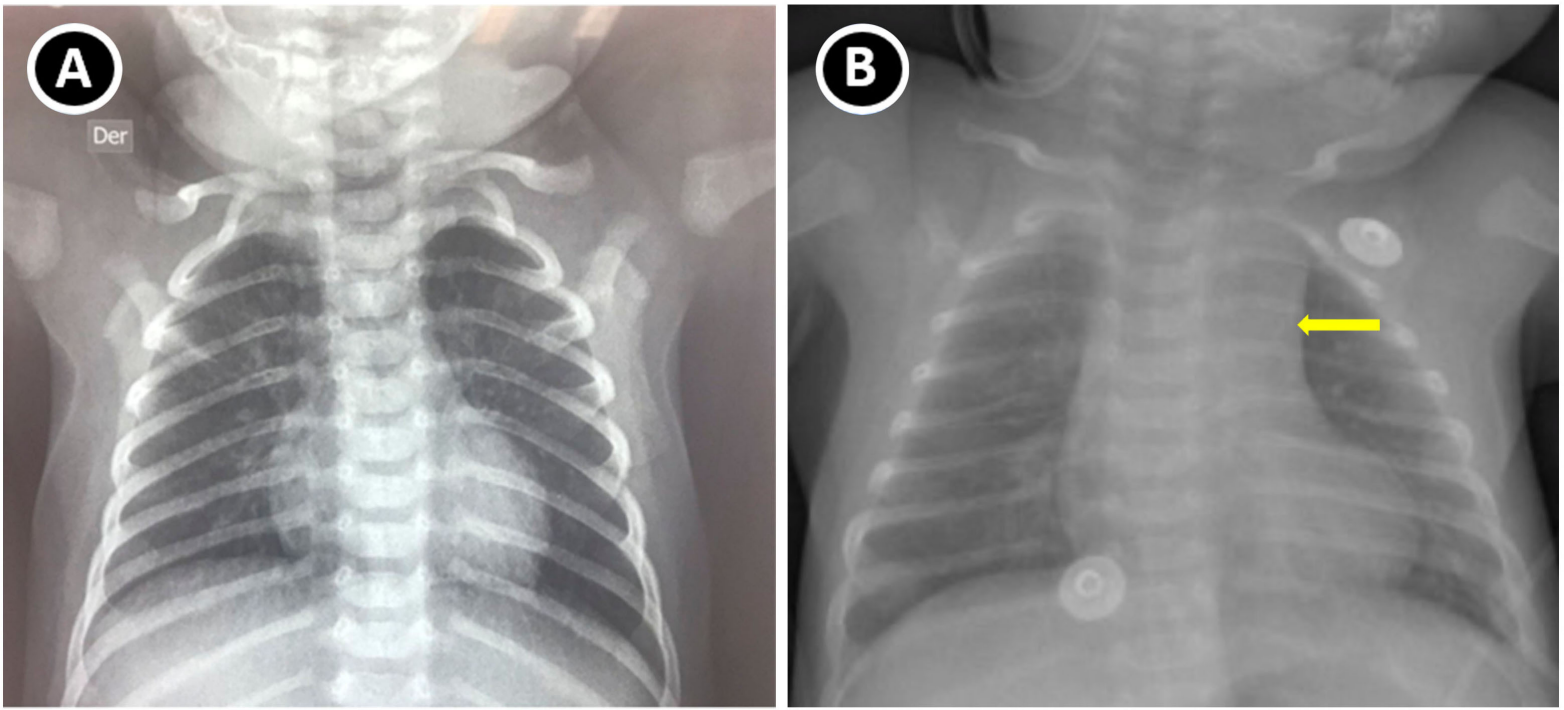
Chest radiography. **(A)** Chest radiograph of patient P2 showing absence of the thymic shadow, consistent with loss of the anterior mediastinal contour. **(B)** Normal control with preserved thymic silhouette (yellow arrow).

Chest radiography demonstrated absence of the thymic silhouette (**Figure 4**). Prophylaxis with TMP–SMX 5 mg/kg was started every third day, acyclovir 12.5 mg/kg/day, fluconazole 5 mg/kg/day, and IVIG 800 mg/kg/month, being asymptomatic.

At three months of age, he experienced a single self-limiting diarrheal episode lasting 24 hours. At 5 months, molecular analysis by Next Generation Sequencing (NGS) at an external reference laboratory was made according to the following methodology. Whole Exome Sequencing (WES) was performed using the Ion Torrent AmpliSeq RDY Exome Kit (Life Technologies) and the Ion Chef and Proton instruments (Life Technologies). Briefly, 100 ng of gDNA was used as the starting material for the AmpliSeq RDY Exome amplification step following the manufacturer’s protocol. Library templates were clonally amplified and enriched using the Ion Chef and the Ion PI Hi-Q Chef Kit (Chef package version IC.4.4.2, Life Technologies) and then sequenced on the Ion Proton using the Ion PI chip v3 (Life Technologies). Read mapping and variant calling were performed using the Ion Torrent Suite software v4.4.2. Variants were annotated using ANNOVAR (http://annovar.openbioinformatics.org/). For Sanger sequencing of IL2RG(NM_000206):c.667G>T, p.(V223F), genomic DNA was PCR-amplified using GoTaq DNA Polymerase (Promega) and variant-flanking, M13-tailed oligonucleotide primers as follows: forward: 5’-TGTAAAACGACGGCCAGTcactggtgggtgttcaggagtatgtttta-3’ and reverse: 5’-CAGGAAACAGCTATGACCagtggtgttagaaaggctggggtgttg-3’. PCR products were purified using ExoSAP-IT (Thermo Fisher Scientific) and were directly sequenced using BigDye Terminators (v.1.1) and universal M13 forward and reverse primers. Sequencing products were analyzed using a 3500xL Genetic Analyzer (Thermo Fisher Scientific).A hemizygous *IL2RG* variant c.667G>T; (p.V223F) was identified and confirmed a G to T transition at nucleotide position 667 in exon 6, resulting in a Valin (V) to Phenylalanine (F) change at codon 223. Human leukocyte antigen (HLA) typing was performed for the patient and family members; however, no compatible donor was identified, and hematopoietic stem cell transplantation (HSCT) was not feasible at our institution at that time. Consequently, the patient met eligibility criteria for gene therapy within an ongoing clinical trial at St. Jude Children’s Research Hospital and was referred for treatment; but, upon arrival, he was found to have an adenovirus infection, which became complicated and ultimately led to his death. The timeline of events is shown in **Figure 3B**.

## 2 Discussion

X-SCID is the most common form of SCID, accounting for approximately 30% of cases and typically characterized by a T−B+NK− phenotype (9). Consanguinity is a well-recognized factor contributing to the increased prevalence of many autosomal recessive IEI (10), It is important to clarify that *IL2RG* follows an X-linked inheritance pattern. In this case, the maternal carrier status determines the risk for the male siblings, and the consanguinity within the pedigree, although noted, does not alter the transmission dynamics of this specific X-linked trait. This distinction is vital for accurate genetic counseling in X-SCID families.

In Mexico, the high prevalence of tuberculosis supports the routine administration of BCG vaccination as a preventive strategy (11). However, in infants with SCID, this practice carries substantial risk because immunization typically occurs before the underlying immunodeficiency is recognized. It has been estimated that up to 51% of affected patients may develop disseminated complications involving primarily the lymph nodes, skin, and lungs, and less frequently the spleen, liver, and bones (12). In P2, these complications were prevented because vaccination was withheld in light of the family history.

The *IL2RG* variant c.667G>T (p.V223F) was submitted to ClinVar in 2018 and classified as “likely pathogenic” due to limited clinical data and its absence in large population databases (13). However, the clinical progression observed in this family provides robust evidence for its reclassification to “pathogenic” based on the ACMG standards and guidelines.

Specifically, the occurrence of the same severe T−B+NK− phenotype in two male siblings from a consanguineous pedigree fulfills the PP1-Strong criterion (co-segregation with disease in multiple affected family members). Furthermore, the fatal outcome and the severe disseminated BCG infection observed in our patients serve as strong clinical indicators of the variant’s complete penetrance and high virulence, satisfying the PS3 (strong benign/pathogenic functional evidence) as the clinical phenotype perfectly matches the established pathophysiology of X-SCID. When combined with the existing PM2 criterion (absence in healthy populations) and PP3 (in silico computational predictions favoring a deleterious effect), the aggregate evidence definitively supports a pathogenic status (8). This reclassification is crucial for future genetic counseling and emphasizes that p.V223F should be treated as a high-risk mutation requiring immediate HSCT upon detection.

**Figure 5:**
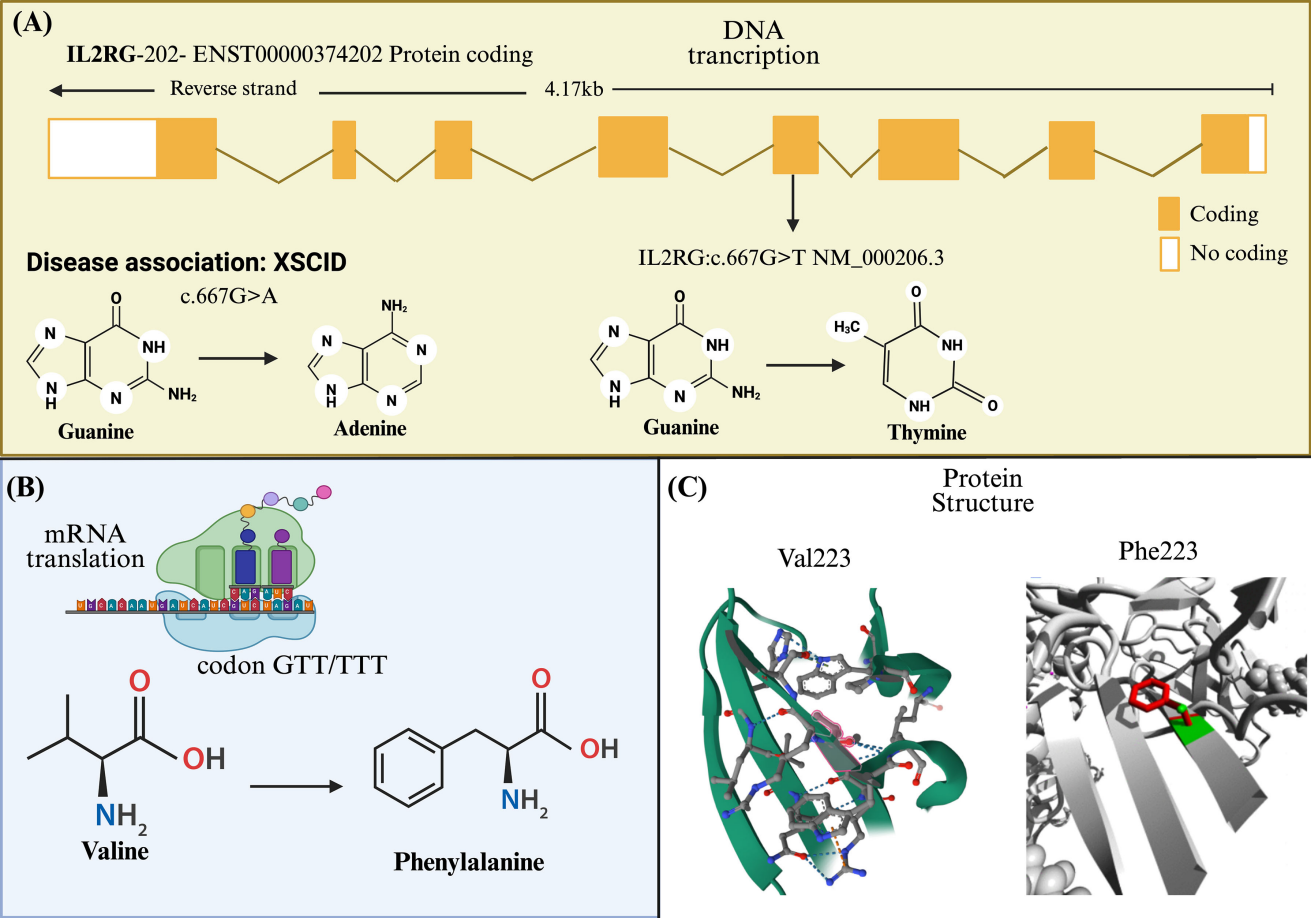
Overview of the structure and protein translation of *the IL2RG* gene. **(A)** Gene structure of *IL2RG* (ENST00000374202) located on the reverse strand. The identified nucleotide change, c.667G>T (NM_000206.3), results in a substitution of guanine to thymine. Notably, a different variant at the same position, c.667G>A (changes different amino acid), has been reported in ClinVar as associated with XSCID; however, its specific clinical characteristics have not yet been defined. **(B)** Translation of mRNA showing the resulting missense mutation p.Val223Phe (NP_000197.1), where valine is replaced by phenylalanine. **(C)** Protein structural model that highlights the mutation site. The panel displays the wild-type residue Val223 (highlighted in red, left side) and, to the right, the mutated amino acid Phe223 (in red), illustrating the potential structural impact of the substitution on the γc chain, which may lead to altered protein folding and function. Created by BioRender. Bravo, K (2025). https://BioRender.com/eyyus07.

The classification of the c.667G>T (p.V223F) variant as pathogenic is supported by a multi-layered analysis of protein structure UniProt platform (14), bioinformatic prediction (**Figure 5**), and clinical correlation. Although the variant is located within a region associated with specific chromatin spatial organizations (TADs), its primary impact is likely driven by the amino acid substitution within the γc (15).

Analysis via the HOPE bioinformatics platform (16) (**Figure 5**) indicates that the p.V223F mutation introduces a phenylalanine residue, which possesses different physicochemical properties compared to the wild-type valine. This change occurs in a critical domain of the IL2RG protein, potentially disrupting folding and abolishing its signaling function. This is further corroborated by a MetaRNN score of 0.904 (range: 0.0-1.0) from the dbNSFP platform, a value that strongly indicates pathogenicity based on deep learning integration of multiple conservation and functional scores (17).

Crucially, our study provides the missing genotype-phenotype correlation required for reclassification. The clinical presentation of our patients aligns with previous reports of nearby missense variants, such as p.A222C (18) and p.L293G (19). All cases share a consistent T−B+NK− immunophenotype, suggesting a common molecular mechanism of immune disruption. By documenting the fatal clinical outcome and severe complications in this pedigree, we fulfill the PS3 (Strong clinical/functional evidence) and PP1 (Co-segregation) criteria of the ACMG guidelines. Consequently, these findings justify upgrading the p.V223F variant from “likely pathogenic” to “pathogenic”, providing a clearer prognostic framework for future clinical management.

Genetic testing to distinguish pathogenic variants differentiates SCID from athymia and is essential for a precise molecular diagnosis, facilitating the choice of an optimal transplantation plan and allowing enzyme replacement therapy in patients with adenosine deacetylase (ADA) deficiency. Furthermore, genetic defects can be identified, genetic counseling and family planning can be provided, but it is not a prerequisite to begin treatment given the severity of the disease (20).

Quantification of T cell receptor excision circles (TREC) is applied in newborn screening (NBS) using dried blood spot (DBS) samples collected on Guthrie cards to detect SCID (21). In Turkey, a pilot study conducted between 2018 and 2020 identified two infants with undetectable TREC levels who were later diagnosed with SCID. Genetic analysis confirmed defects in *ADA* and *RAG1*, and both patients received curative treatment (21). Similarly, in Brazil, newborns screened between 2009 and 2020 with low TREC levels included 24 SCID cases, of whom 66.7% underwent HSCT, with a success rate of 70% (20). These programs highlight the importance of early detection and timely treatment, which improve survival and quality of life; however, such initiatives have not yet been implemented in Mexico.

## 3 Conclusions

This study provides the clinical and molecular evidence required to reclassify the *IL2RG* c.667G>T (p.V223F) variant from “likely pathogenic” to “pathogenic”. By documenting the severe immunological phenotype and fatal outcomes observed in this family, we establish a clear genotype–phenotype correlation that reinforces the diagnostic framework for X-SCID.

Our findings underscore that, in the absence of universal NBS, early molecular diagnosis and a thorough assessment of family history are critical to ensuring timely access to life-saving interventions. Ultimately, this report contributes to the global genetic landscape of IEI and highlights the vital role of variant reclassification in improving genetic counseling and clinical outcomes for affected families.

## 4 Limitations

While this report provides critical evidence for the classification of the p.V223F variant, certain limitations must be acknowledged. First, our findings are based on a single familial cluster; although the clinical correlation is strong, a broader cohort would further validate the phenotypic spectrum of this mutation. Second, genetic analysis was performed using NGS at an external reference laboratory. Detailed information regarding sequencing validation procedures, quality control measures, and the analytical workflow was not available to the authors and therefore could not be fully described. Third, while bioinformatic tools and clinical outcomes strongly suggest pathogenicity, *in vitro* functional assays were not performed to definitively characterize the impact on **γc** signaling. Lastly, due to logistical constraints, segregation analysis was limited to the index cases and their immediate pedigree, preventing a broader genomic mapping of the extended family.

## Data Availability

The original contributions presented in the study are includedin the article/**Supplementary material**. For any further questions, please contact the corresponding author and request any information you may need regarding the manuscript.

## References

[1] Dalal I . Tasher. In: The genetic basis of severe combined immunodeficiency and its variants, vol. 67. TACG (2012). 10.2147/TACG.S18693 PMC3681194 23776382

[2] Jacovas VC Zelnick M McNulty S Ross JE Khurana N Pan X . The ClinGen Severe Combined Immunodeficiency Disease Variant Curation Expert Panel: Specifications for classification of variants in ADA, DCLRE1C, IL2RG, IL7R, JAK3, RAG1, and RAG2. Genet Med. (2026) 28:101613. doi: 10.1016/j.gim.2025.101613, PMID: 41104538 PMC13175239

[3] Lin JX Leonard WJ . The common cytokine receptor γ Chain family of cytokines. Cold Spring Harb Perspect Biol. (2018) 10:a028449. doi: 10.1101/cshperspect.a028449, PMID: 29038115 PMC6120701

[4] Kwan A Abraham RS Currier R Brower A Andruszewski K Abbott JK . Newborn screening for severe combined immunodeficiency in 11 screening programs in the United States. JAMA. (2014) 312:729. doi: 10.1001/jama.2014.9132, PMID: 25138334 PMC4492158

[5] Aranda CS Gouveia-Pereira MP Da Silva CJM Rizzo MCFV Ishizuka E De Oliveira EB . Severe combined immunodeficiency diagnosis and genetic defects. Immunol Rev. (2024) 322:138–47. doi: 10.1111/imr.13310, PMID: 38287514

[6] Notarangelo LD . Genetically-determined defects of T cell development. Allergy Asthma Proc. (2024) 45:326–31. doi: 10.2500/aap.2024.45.240028, PMID: 39294907 PMC11425799

[7] George K Govindaraj G . Infections in inborn errors of immunity with combined immune deficiency: A review. Pathogens. (2023) 12:272. doi: 10.3390/pathogens12020272, PMID: 36839544 PMC9958715

[8] Richards S Aziz N Bale S Bick D Das S Gastier-Foster J . Standards and guidelines for the interpretation of sequence variants: a joint consensus recommendation of the American College of Medical Genetics and Genomics and the Association for Molecular Pathology. Genet Med. (2015) 17:405–24. doi: 10.1038/gim.2015.30, PMID: 25741868 PMC4544753

[9] Ravichandran KS Burakoff SJ . The adapter protein Shc interacts with the interleukin-2 (IL-2) receptor upon IL-2 stimulation. J Biol Chem. (1994) 269:1599–602. doi: 10.1016/S0021-9258(17)42066-7 8294403

[10] Aykut A Durmaz A Karaca N Gulez N Genel F Celmeli F . Severe combined immunodeficiencies: Expanding the mutation spectrum in Turkey and identification of 12 novel variants. Scand J Immunol. (2022) 95:e13163. doi: 10.1111/sji.13163, PMID: 35303369

[11] Escobar-Gutierrez A Martinez-Guarneros A Mora-Aguilera G Vazquez-Chacon CA Acevedo-Sanchez G Sandoval-Díaz M . The First exploratory spatial distribution analysis of tuberculosis and associated factors in Tonala, Mexico. J Infect Dev Ctries. (2020) 14:207–13. doi: 10.3855/jidc.11873, PMID: 32146456

[12] Marciano BE Huang CY Joshi G Rezaei N Carvalho BC Allwood Z . BCG vaccination in patients with severe combined immunodeficiency: Complications, risks, and vaccination policies. J Allergy Clin Immunol. (2014) 133:1134–41. doi: 10.1016/j.jaci.2014.02.028, PMID: 24679470 PMC4015464

[13] Ambry’s Exome Reporting Categories . NM_000206.3(IL2RG):c.667G>T (p.Val223Phe) AND Inborn genetic diseases - ClinVar - NCBI. ClinVar - NCBI (2018). Available online at: https://www.ncbi.nlm.nih.gov/clinvar/RCV001267195/.

[14] Ahmad S da Costa Gonzales LJ Bowler-Barnett EH Rice DL Kim M Wijerathne S . UniProt. UniProt Consortium The UniProt website API (2025). Available online at: https://www.uniprot.org/help/publications (Accesed February 9, 2025).

[15] McArthur E Capra JA . Topologically associating domain boundaries that are stable across diverse cell types are evolutionarily constrained and enriched for heritability. Am J Hum Genet. (2021) 108:269–83. doi: 10.1016/j.ajhg.2021.01.001, PMID: 33545030 PMC7895846

[16] Venselaar H Te Beek TA Kuipers RK Hekkelman ML Vriend G . Protein structure analysis of mutations causing inheritable diseases. An e-Science approach with life scientist friendly interfaces. BMC Bioinf. (2010) 11:548. doi: 10.1186/1471-2105-11-548, PMID: 21059217 PMC2992548

[17] Li C Zhi D Wang K Liu X . MetaRNN: differentiating rare pathogenic and rare benign missense SNVs and inDels using deep learning. Bioinformatics. (2021) 14:115. doi: 10.1101/2021.04.09.438706, PMID: 36209109 PMC9548151

[18] Sharfe N Shahar M Roifman CM . An interleukin-2 receptor gamma chain mutation with normal thymus morphology. J Clin Invest. (1997) 100:3036–43. doi: 10.1172/JCI119858, PMID: 9399950 PMC508516

[19] Schmalstieg FC Leonard WJ Noguchi M Berg M Rudloff HE Denney RM . Missense mutation in exon 7 of the common gamma chain gene causes a moderate form of X-linked combined immunodeficiency. J Clin Invest. (1995) 95:1169–73. doi: 10.1172/JCI117765, PMID: 7883965 PMC441454

[20] Barreiros LA Sousa JL Geier C Leiss-Piller A Kanegae MPP França TT . SCID and other inborn errors of immunity with low TRECs — the Brazilian experience. J Clin Immunol. (2022) 42:1171–92. doi: 10.1007/s10875-022-01275-9, PMID: 35503492

[21] Haskologlu S Kocak S Tufan LS Aksoy FE Bastug D Oner DA . Newborn screening for SCID: the very first prospective pilot study from Türkiye. Front Immunol. (2024) 15:1384195. doi: 10.3389/fimmu.2024.1384195, PMID: 39483481 PMC11526446

