# Supplementary material for: X-linked severe combined immunodeficiency due to IL2RG p.V223F variant: clinical evidence that support its pathogenicity- a case report
Source: Front Immunol. 2026 Apr 14;17:1776191. doi: 10.3389/fimmu.2026.1776191 (PMC13120896; doi:10.3389/fimmu.2026.1776191)
Supplement: Supplementary file 1 [file DataSheet1.pdf]

2013 CARE Checklist

1. **Title**: X-Linked Severe Combined Immunodeficiency due to *IL2RG* p.V223F Variant: Clinical evidence that support its pathogenicity- a case report.
2. **Key Words**: severe combined immunodeficiency, IL2RG gene, X-linked severe combined immunodeficiency, c.667 G>T , p.V223F, s2092258151, case report.
3. **Abstract**: This report details two male siblings from a consanguineous union (first cousins) diagnosed with X-linked Severe Combined Immunodeficiency (X-SCID) with a $T^{-}B^{+}NK^{-}$ immunophenotype. The first patient (P1) presented at one month of age with persistent cough after the BCG vaccine, progressing to disseminated BCGitis (suggestive of tuberculosis), diarrhea, and weightloss. Immunological evaluation confirmed profound T-cell lymphopenia and undetectable IgA/IgM, leading to a diagnosis of SCID. P1 died at 12 months of septic shock. The second patient (P2) was immunologically evaluated early due to the family history, showing profound T cell lymphopenia with the same immunophenotype. Molecular analysis identified a hemizygous variant (c.667G>T; p.V223F). P2 died at 8 months of severe pneumonia. The cases highlight the contribution of consanguinity to inborn errors of Immunity (IEI) and the risk of BCG vaccination in undiagnosed SCID patients in endemic regions such as Mexico. The report contributes clinical and genetic information to support the reclassification of the IL2RG variant as pathogenic.
4. **Introduction**: Severe combined immunodeficiency (SCID) is an inherited disorder resulting from genetic defects in the immune system. It represents a heterogeneous group of conditions characterized by impaired development and function of T and B lymphocytes, often with additional involvement of natural killer (NK) cells, rendering affected individuals highly susceptible to infections (Tasher D *et al*, 2012). SCID is recognized as one of the most severe forms of inborn errors of immunity (IEI), with an estimated incidence of 1 in 58,000 live births (Kwan A *et al*, 2014). Without timely and accesible treatment, most affected children do not survive beyond the first two years of life (Aranda CS *et al*, 2024).

   SCID is diverse in its presentation and may manifest as typical or leaky variants, depending on the severity of quantitative and qualitative T-cell deficiency. These immune defects disrupt both adaptive and celular immune mechanisms, leaving affected individuals vulnerable to a wide spectrum of pathogens (Notarangelo LD, 2024). Importantly, the clinical manifestations of SCID can vary considerably, as mutations in the same gene may result in different degrees of immune dysfunction depending on penetrance, the specific functional defect involved, and the variable expressivity of the affected gene (George K *et al*, 2023).

   In clinical practice, it has been observed that the most common genetic cause of SCID is variants in IL2RG (X-linked SCID), accounting for 30% of cases (Vanessa CJ *et al*, 2025). IL2RG encodes the common gamma chain (γc) of the interleukin-2 receptor. Since the γc chain is also required for signaling through the receptors of IL-4, IL-7, IL-9, IL-15, and IL-21, its disruption results in defective development of T cells and NK cells, while B cells are present but functionally impaired. Consequently, affected patients typically exhibit a $T^{-}B^{+}NK^{-}$ immunophenotype (Lin JX *et al*, 2018).

   This immunologic profile is strongly associated with severe, recurrent infections and poor responses to vaccination, making the relationship between SCID and

IL2RG variants a critical consideration for diagnosis. Here, we describe two male siblings born to consanguineous parents who presented with X-linked SCID caused by a previously reported IL2RG variant (c.667G>T; p.V223F). Both patients displayed the characteristic $T^{-}B^{+}NK^{-}$ phenotype and experienced severe infectious complications leading to early mortality. This report highlights the clinical, immunologic, and genetic findings of the affected siblings and underscores the ongoing challenges of diagnosis and management in resource-limited settings where curative therapies may not be readily available.

5. **Patient Information:**

   - De-identified patient specific information.

   P1: Male infant, product of a third pregnancy. Parents are fourth-degree relatives (first cousins).

   P2: Male infant, product of a fifth pregnancy (preceded by an intrauterine death). Parents are the same.

   - Primary concerns and symptoms of the patient.

   P1: Persistent cough, diarrhea, weightloss, swollen and draining BCG inoculation site, hyperpigmented subcutaneous nodules, signs of increased work of breathing (retractions, tachypnea).

   P2: None initially. Family history prompted early screening. Later, a single, self-limiting diarrheal episode.

   - Medical, family, and psychosocial history including relevant genetic information.

   Parents: Fourth-degree relatives (first cousins). Mother (25) and Father (31) were healthy. Family History: Notable for early mortality from infectious diseases. Two maternal uncles died at 6 and 8 months from diarrheal diseases. P1's older brother (the third child, III-3) died at 12 months from septic shock. Genetic Information: P2 was found to have a hemizygous variant of the *IL2RG* gene (c.667G>T; p.V223F), consistent with X-linked SCID.

   - Relevant past interventions and their outcomes.

   P1: Received BCG vaccine at 1 month, which led to complications (disseminated BCGitis/Tuberculosis).

   P2**:** No immunizations were administered due to family history. Breastfeeding was discontinued because the mother was CMV positive.

6. **Clinical Findings**: P1: Hypoactive, multiple firm, non-tender subcutaneous nodules (0.5–1.0 cm) in the arms and abdomen. Grade II holosystolic murmur. Vesicular breath sounds with coarse features. Signs of increased work of breathing (intercostal and xiphoid retractions, tachypnea). P2: The physical examination findings were not notable. The chest radiography revealed the absence of the thymic silhouette.

7. **Timeline**:

Age	P1 Events	P2 Events
**1 Month**	BCG Vaccination; develop persistent cough.	Immunological evaluation: deep T-cell lymphopenia (SCID) due to family history. Prophylaxis started
**3 Months**	Diarrhea, wheezing cough, weightloss.	Single self-limiting diarrheal episode (24 h).
**5 Months**	Swollen BCG site, purulent material; subcutaneous nodules appeared.	Molecular analysis identified the IL2RG variant.
**8 Months**	-	Died of severe pneumonia-related complications.
**9 Months**	The biopsy of the lesions suggested tuberculosis. Cytopenias noted. Referred for evaluation.	-
**12 Months**	Died from septic shock.	-

8. **Diagnostic Assessment:**

- Diagnostic methods (PE, laboratory testing, imaging, surveys). P1: PE (nodules, respiratory distress), Complete Blood Count (cytopenias), Biopsy (chronic granulomatous inflammation, Acid-Fast Bacilli, Salmonella spp.), Immunological Evaluation (Profound T cell lymphopenia: CD3: 14 cells/µL; Undetectable IgA/IgM), SCID $T^{-}B^{+}NK^{-}$ immunophenotype. P2: Immunological Evaluation (Profound T cell lymphopenia: CD3: 0 cells/µL), Chest X-ray (absence of thymic silhouette), Molecular Analysis (IL2RG variant).
- Diagnostic challenges. P1: Initial presentation was complicated by severe opportunistic infections (Disseminated BCG/Tuberculosis and Salmonellosis), which masked the underlying immunodeficiency until profound lymphopenia was noted. P2: While the diagnosis of SCID was made early, genetic testing was necessary to distinguish the precise molecular defect and guide treatment (HSCT).
- Diagnosis (including other diagnoses considered). Primary Diagnosis: Severe Combined Immunodeficiency (SCID) with $T^{-}B^{+}NK^{-}$ immunophenotype, confirmed as X-linked SCID in P2 by the IL2RG variant. Secondary/Complicating Diagnoses (P1): Disseminated Tuberculosis (BCGitis), Salmonellosis.
- Prognostic characteristics when applicable. Profound T-cell lymphopenia and undetectable immunoglobulin levels indicate a severe prognosis without intervention. The diagnosis of SCID without timely treatment (like HSCT) leads to death within the first year of life.

9. Therapeutic Intervention:
    - Types of therapeutic intervention (pharmacologic, surgical, preventive).
    - P1: He received treatment for tuberculosis with rifampicin10 mg/kg/day, isoniacid 10 mg/kg/day, and ethambutol 30 mg/kg/day; antifungals with

fluconazole 6 mg/kg/day, antibiotics with ciprofloxacin 30 mg/kg/day, and ticarcillin/clavulanic acid 200 mg/kg/day; prophylaxis with trimethoprim-sulfamethoxazole (TMP–SMX) 5 mg/kg every third day, ganciclovir 10 mg/kg/day, as well as intravenous immunoglobulin (IVIG) replacement therapy 800 mg/kg/month.

- P2: Prophylaxis with TMP–SMX 5 mg/kg every third day, acyclovir 12.5 mg/kg/day, fluconazole 5 mg/kg/day, and intravenous immunoglobulin (IVIG) 800 mg/kg/month was started.
- Changes in therapeutic interventions with explanations. The primary change was the initiation of an Hematopoietic Stem Cell Transplantation (HSCT) protocol for both patients, which is the curative treatment for SCID. In P1 A hematopoietic stem cell transplantation (HSCT) protocol was initiated; however, the patient unfortunately died at 12 months of age from septic shock. For P2 HLA typing was performed for the patient and family members, but no compatible donor was available, and HSCT was not feasible at the hospital at that time. The patient therefore met the eligibility criteria for gene therapy in an ongoing clinical trial at St. Jude Children's Research Hospital targeting this pathogenic variant. He was accepted and referred for treatment; however, upon arrival, he was found to have an adenovirus infection, which became complicated and ultimately led to his death.

10. **Follow-up and Outcomes**

- Clinician- and patient-assessed outcomes if available: P1 had infectious complications: BCGitis and BCGosis, infection caused by *Salmonella spp*. A hematopoietic stem cell transplantation protocol was initiated; however, the patient unfortunately died at 12 months of age.
- P2: He was accepted and referred for gene therapy; however, upon arrival, he was found to have an adenovirus infection, which became complicated and ultimately led to his death.
- Important follow-up diagnostic and other test results: P1: Histological slides confirmed Acid-Fast Bacilli (AFB) and Salmonella spp. The culture of the biopsy specimen identified *Mycobacterium tuberculosis* with resistance to pyrazinamide. P2: Molecular analysis identified the *IL2RG* variant (c.667G>T; p.V223F).
- Intervention adherence and tolerability. (How was this assessed?): Adherence and tolerability are not explicitly detailed, but prophylactic and treatment regimens were started and maintained until death.
- Adverse and unanticipated events. P1: Developed disseminated complications (tuberculosis/BCGitis) following the routine BCG vaccine. Death from septic shock. P2: The patient did not undergo bone marrow transplantation due to HLA incompatibility, and HSCT was not feasible at the hospital at that time.; however, gene therapy was considered viable at St. Jude Children's Research Hospital, and the patient was referred there for this

procedure, but died from an adenovirus infection, which became complicated.

11. Discussion

- Strengths and limitations in your approach to this case: Strengths: The report provides detailed clinical and molecular confirmation of the diagnosis, linking the IL2RG variant to the $T^{-}B^{+}NK^{-}$ phenotype. It adds comprehensive data to the literatura for a previously poorly characterized variant. Early diagnosis was achieved for P2 based on family history. Limitations: The planned HSCT (curative treatment) was unsuccessful/not performed for both patients (P1: death before transplant, P2: HLA incompatibility, lack of resources for HSCT, death before gene therapy), limiting information on the long-term success of treatment. Genetic testing was not a prerequisite for starting treatment, indicating a delay in molecular diagnosis for P1.
- Discussion of the relevant medical literature: Consanguinity: Mentioned as a key determinant for autosomal recessive disorders, with studies in Turkish SCID cohorts documenting a high consanguinity rate (approx. 23.2%) (Aykut A *et al*, 2022). X-SCID: Confirmed as the most common SCID form (50–60% of cases) with the $T^{-}B^{+}NK^{-}$ phenotype (Ravichandran KS, Burakoff SJ, 1994). BCG: Up to 51% of SCID patients develop disseminated complications from BCG, highlighting the risk of vaccinating before diagnosis in countries like Mexico (Marciano BE *et al*, 2014). Genetic Variant: The discussion supports the reclassification of the *IL2RG* variant (p.V223F) to pathogenic based on: a) bioinformatics scores (MetaRNN score of 0.90400016), b) functional prediction (HOPE platform suggesting disruption of domain), and c) positional context within a Topologically Associating Domain (TAD). NBS: Highlighted the importanceof T-cell receptor excision circles (TREC)-based Newborn Screening (NBS) programs (citing success in Turkey and Brazil) for early detection and timely HSCT, noting the absence of such programs in Mexico (Aykut A *et al*, 2022; Barreiros LA et al, 2022).
- The rationale for your conclusions: The severe recurrent infections, profound T-cell lymphopenia, characteristic $T^{-}B^{+}NK^{-}$ immunophenotype, and identification of the hemizygous *IL2RG* variant confirm the diagnosis of X-SCID. The consanguineous relationship of the parents, the death of a previous sibling, and the family history of early mortality from infection strongly support an inherited error of immunity. The complications seen in P1 following BCG underscore the necessity of SCID NBS.
- The primary "take-away" lessons from this case report (without references) in a one paragraph conclusion: The clinical course of these two SCID-affected male siblings from a consanguineous unión emphasizes the critical need for comprehensive family history taking in diagnosing Inborn Errors of Immunity. Despite the most common form of SCID being X-linked, consanguinity may be a contributing factor to the high prevalence of IEI in

certain populations, and this case adds robust evidence for the pathogenicity of a specific *IL2RG* variant. The tragic outcome of the first patient following BCG vaccination, an intervention widely used in high-prevalence settings, serves as a powerful illustration of the urgent need to implement SCID newborn screening programs to allow for early intervention and prevent life-threatening complications.

12. **Patient perspective:** This item does not apply to the report, as the patients were children and both died at an early age.
13. **Informed Consent:** The informed consent was obtained.
